# Repressive H3K27me3 drives hyperglycemia-induced oxidative and inflammatory transcriptional programs in human endothelium

**DOI:** 10.1186/s12933-024-02196-0

**Published:** 2024-04-05

**Authors:** Julia Sánchez-Ceinos, Shafaat Hussain, Abdul Waheed Khan, Liang Zhang, Wael Almahmeed, John Pernow, Francesco Cosentino

**Affiliations:** 1 Cardiology Unit, Department of Medicine-Solna, Karolinska Institutet, Karolinska University Hospital, Stockholm, Sweden; 2 Department of Molecular and Clinical Medicine, University of Gothenburg, Gothenburg, Sweden; 3 Department of Diabetes, Central Clinical School, Monash University, Melbourne, Australia; 4 Heart and Vascular Institute, Cleveland Clinic Abu Dhabi, Abu Dhabi, UAE

**Keywords:** Diabetes, Epigenetics, Endothelial cells, Oxidative stress, Inflammation, Chromatin-modifying drugs, EZH2 inhibitor GSK126

## Abstract

**Background:**

Histone modifications play a critical role in chromatin remodelling and regulate gene expression in health and disease. Histone methyltransferases EZH1, EZH2, and demethylases UTX, JMJD3, and UTY catalyse trimethylation of lysine 27 on histone H3 (H3K27me3). This study was designed to investigate whether H3K27me3 triggers hyperglycemia-induced oxidative and inflammatory transcriptional programs in the endothelium.

**Methods:**

We studied human aortic endothelial cells exposed to high glucose (HAEC) or isolated from individuals with diabetes (D-HAEC). RT-qPCR, immunoblotting, chromatin immunoprecipitation (ChIP-qPCR), and confocal microscopy were performed to investigate the role of H3K27me3. We determined superoxide anion (O_2_^−^) production by ESR spectroscopy, NF-κB binding activity, and monocyte adhesion. Silencing/overexpression and pharmacological inhibition of chromatin modifying enzymes were used to modulate H3K27me3 levels. Furthermore, isometric tension studies and immunohistochemistry were performed in aorta from wild-type and *db/db* mice.

**Results:**

Incubation of HAEC to high glucose showed that upregulation of EZH2 coupled to reduced demethylase UTX and JMJD3 was responsible for the increased H3K27me3. ChIP-qPCR revealed that repressive H3K27me3 binding to superoxide dismutase and transcription factor JunD promoters is involved in glucose-induced O_2_^−^ generation. Indeed, loss of JunD transcriptional inhibition favours NOX4 expression. Furthermore, H3K27me3-driven oxidative stress increased NF-κB p65 activity and downstream inflammatory genes. Interestingly, EZH2 inhibitor GSK126 rescued these endothelial derangements by reducing H3K27me3. We also found that H3K27me3 epigenetic signature alters transcriptional programs in D-HAEC and aortas from *db/db* mice.

**Conclusions:**

EZH2-mediated H3K27me3 represents a key epigenetic driver of hyperglycemia-induced endothelial dysfunction. Targeting EZH2 may attenuate oxidative stress and inflammation and, hence, prevent vascular disease in diabetes.

**Graphical Abstract:**

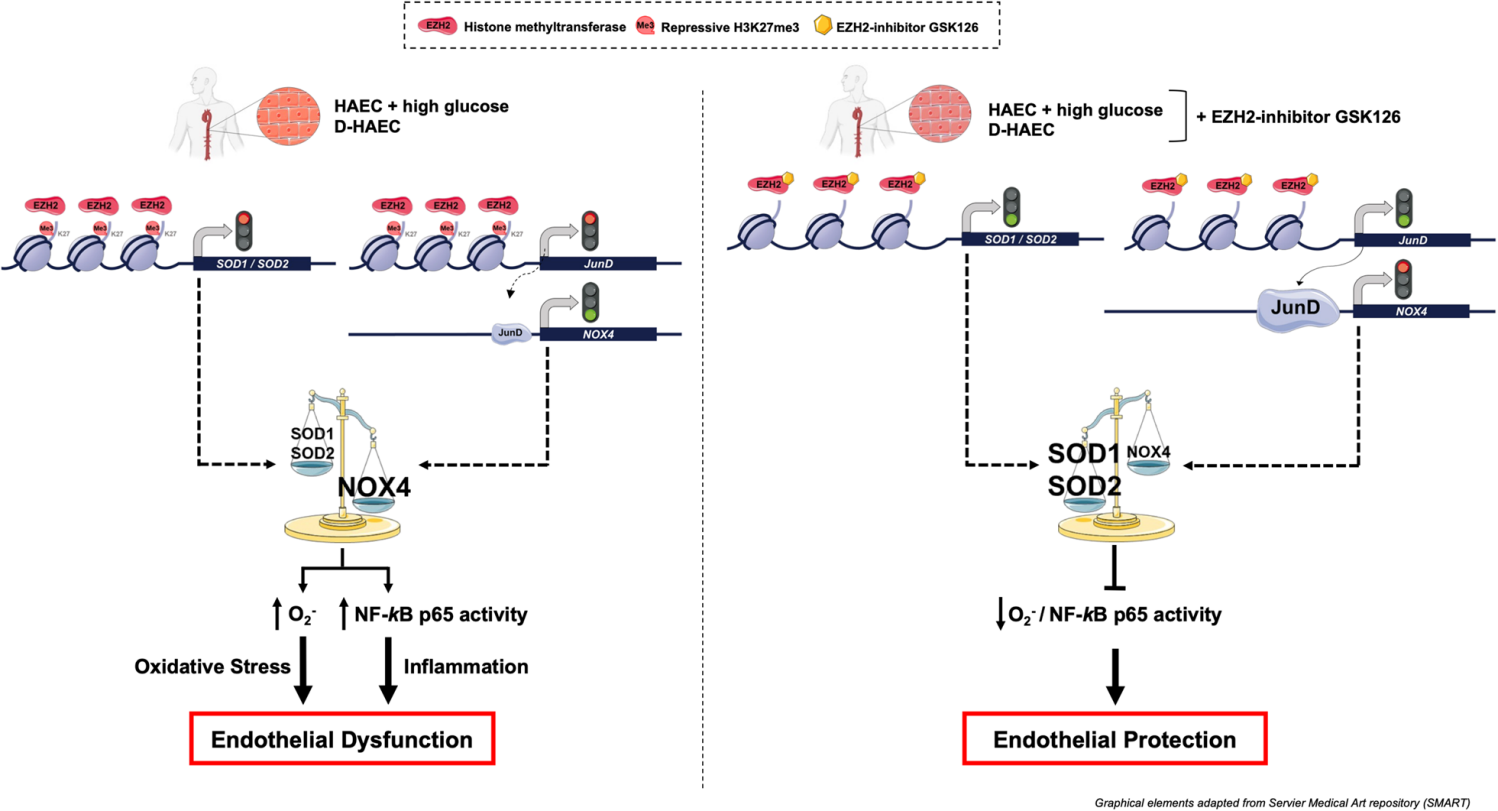

**Supplementary Information:**

The online version contains supplementary material available at 10.1186/s12933-024-02196-0.

## Background

The worldwide prevalence of diabetes is exceptionally high, and the number of patients is expected to rise to 700 million by 2045 [1]. Cardiovascular disease represents the leading cause of morbidity and mortality in this setting [2]. Endothelial dysfunction and inflammation are key hallmarks of the adverse diabetic vascular phenotype [3]. Although a large body of evidence has revealed that excessive production of reactive oxygen species (ROS) precedes their development [4] the molecular drivers of oxidative and inflammatory transcriptional programs remain to be fully elucidated.

Epigenetic changes are emerging as important players in cardiovascular disease [5]. Indeed, alterations of the epigenome leading to plastic modifications of chromatin organization affect gene expression [6]. A crucial mechanism that regulates chromatin architecture is the covalent modification of specific amino residues on histone tails [7]. Different histone modification patterns (e.g., methylation or acetylation) change chromatin accessibility [8]. Histone lysine methylation has been implicated in gene activation and repression, depending on the specific lysine (K) residue that becomes methylated and the state of methylation (mono-, di-, or trimethylation). In particular, trimethylation of lysine 27 on histone 3 (H3K27me3) favours the shift towards heterochromatin, characterized by condensed and silent genomic regions [9]. Polycomb repressive complex 2 (PRC2) is a major chromatin-modifier and plays a role in safeguarding cellular identity by ensuring proper gene silencing [10]. PRC2 comprises multiple core subunits, including enhancer of zeste homolog 1 and 2 (EZH1, EZH2) [11]. EZH2, the main catalytic subunit of PRC2, is a histone methyltransferase that regulates gene expression via H3K27me3 [12].

The methylation on K27 of histone H3 is reversible and can be removed by site-specific histone demethylases UTX, JMJD3, and UTY [12]. It is well established that EZH2 induces such transcriptionally repressive mark in a wide range of genes essential for embryonic development [12]. Aberrant EZH2 activity not only disrupts embryonic development but also impairs tissue homeostasis contributing to cancer, kidney, neurological, metabolic, and cardiovascular diseases [13]. Multiple lines of evidence suggest involvement of EZH2 in atherogenesis [14]. However, its precise role in this setting remains controversial [15–20]. A selective increase of H3K27me3 levels has been found in endothelial cells isolated from human atherosclerotic plaques suggesting that endothelium-specific H3K27me3 enrichment may be important in the development of atherosclerosis [21]. Whether EZH2-induced H3K27me3 is involved in the abnormal vascular phenotypes observed in the presence of diabetes remains to be elucidated. Several small molecules that suppress the enzymatic activity of EZH2 have been recently developed. Clinical trials have reported on their safety and efficacy in different settings [22]. Among others, GSK126 (GSK-2816126) is a highly selective inhibitor of EZH2 methyltransferase activity [23]. The present study was designed to investigate whether inhibition of EZH2-driven H3K27me3 signature with GSK126 blocks adverse transcriptional programs triggered by elevated glucose. Our results unmask a novel mechanism and a druggable target against hyperglycaemia-induced endothelial oxidative stress and inflammation.

## Methods

### Cell culture

Human aortic endothelial cells from healthy individuals (HAEC) and from patients with diabetes (D-HAEC) were purchased from Lonza (Basel, Switzerland). The cells were grown in fibronectin-coated 75 cm^2^ flasks with endothelial cell basal growth media-2 (EBM2; Lonza) supplemented with EGM-2 microvascular endothelial SingleQuotsTM kit (Lonza) and 10% fetal calf serum (FCS; Gibco; Waltham, MA, USA). By using trypsin–EDTA solution (Sigma-Aldrich; Saint Louis, MI, USA) for 3 min, cells were detached and reseeded in fibronectin-coated cell culture dishes. HAEC were cultured in EGM-2 containing 2% FCS and exposed for 20 h either to normal (5 mmol/l) or high glucose (25 mmol/l) concentrations.

To test the effect of pharmacological inhibition of methyltransferase EZH2 or NADPH oxidase, HAEC and/or D-HAEC were treated with EZH2 methyltransferase inhibitor GSK126 (0.5–15 µmol/l; GlaxoSmithKline; Brentford, UK) or NADPH oxidase inhibitor apocynin (100 µmol/l; Sigma-Aldrich; St. Louis, MI, USA), respectively. Treatments with corresponding vehicles (DMSO and ethanol, respectively) were used as control experiments. Since GSK126 (5 µmol/l) did not affect cell viability (Additional file 1: Fig. S1), we used this concentration throughout all the experiments.

### Cell viability assay

Cell viability of HAEC treated with GSK126 was assessed by a commercially available MTT assay kit (Millipore; Burlington, MA, USA). Briefly, cells were exposed for 20 h with increasing concentrations of GSK126 0.5–15 µmol/l. MTT solution (100 µl) was added followed by incubation at 37 ºC for 4 h. Then, isopropanol with 0.04 N HCl was added for colour development. Absorbance was measured on a VersaMax microplate reader (Molecular Devices; San José, CA, USA) at a test and reference wavelength of 570 nm and 630 nm, respectively.

### EZH2 silencing, and UTX and JMJD3 overexpression

HAEC were transfected with targeted double-stranded siRNA oligonucleotides against methyltransferase EZH2 or its negative control (Thermo Fisher Scientific; Waltham, MA, USA) for silencing studies. HAEC were also transfected with a plasmid vector coding for UTX (pCMV-HA-UTX; ID: 24168), JMJD3 (pCMV-HA-JMJD3; ID: 24167), or their corresponding empty vector (pCMV-HA; ID: 32530) (Addgene; Watertown, MA, USA). Transfection was performed using Lipofectamine 2000 Transfection Reagent (Invitrogen; Waltham, MA, USA) following the manufacturer’s instructions. Briefly, the cells were washed with D-PBS (Thermo Fisher Scientific) and submerged into OPTI-MEM medium (Thermo Fisher Scientific) containing 100 nmol/l of the corresponding siRNA or 3 µg of corresponding plasmid vector for 24 h. Thereafter, the transfection medium was removed and a fresh culture medium was added. Finally, after 2 or 3 days of transfection for overexpression or silencing studies, respectively, cells were collected for RT-qPCR, immunoblotting, and ESR spectroscopy analysis. All constructs were verified by DNA sequencing, and transfection efficiency was checked by RT-qPCR and immunoblotting.

### Chromatin immunoprecipitation coupled with quantitative PCR (ChIP-qPCR) assay

Chromatin immunoprecipitation (ChIP) assay was performed in HAEC exposed to either normal or high concentrations of glucose in the presence and in the absence of GSK126 using the Magna ChIP assay kit (Millipore) as described before [24]. HAEC were fixed in 1% of paraformaldehyde (PFA) solution (Santa Cruz Biotechnology; Dallas, TX, USA). Cross-linking was quenched by the addition of 125 mM of glycine (Sigma) for 10 min. After quenching, cells were scraped in D-PBS and centrifuged. Cells were then lysed in SDS lysis buffer and sonicated to obtain chromatin fragments of 200–500 bp using a water bath sonicator (Diagenode; Denville, NJ, USA) 30 s “on”/ 30 s “off” for 15 min. Immunoprecipitation of soluble chromatin was carried out by using specific antibodies against H3K27me3 (Millipore) and JunD (Abcam; Cambridge, UK). Mouse IgG antibody (Abcam) was used as a negative control. Antibody-bound chromatin fractions were precipitated with dynabeads coated with protein A (Invitrogen). Purified DNA sequences were detected by using real-time qPCR (RT-qPCR) and specific primers against *SOD1*, *SOD2*, *JunD*, and *NOX4* promoters (Additional file 1: Table S1). Primers for the promoter region (TSS ± 1000 bp) of each gene were designed using the Oligo Perfect Primer Designer software (Thermo Fisher Scientific). ChIP-qPCR quantifications were performed using the comparative cycle threshold method and reported as the percentage of the antibody-bound chromatin against the total input DNA.

### RNA extraction and real-time (RT-qPCR)

As previously described [25], total RNA was extracted from HAEC and D-HAEC of all experimental groups using TRI Reagent® (Sigma) and Direct-zol™ RNA miniprep kit (Zymo Research; Irvine CA, USA), following the manufacturer’s instructions. Quantification of recovered RNA was assessed using a NanoDrop2000 spectrophotometer (Thermo Fisher Scientific). RNA was retrotranscribed to cDNA using the high-capacity cDNA conversion kit (Applied Biosystems; Foster City, CA, USA). Transcript levels were quantified by RT-qPCR with the FastStart Universal SYBR Green Master Mix (Roche; Basel, Switzerland) on an ABI 7900HT PCR cycler (Applied Biosystems). The cDNA was amplified with a thermal profile at the following conditions: hot-start activation at 95 °C for 10 min, followed by 40 cycles of denaturation (95 °C for 10 s), then annealing/extension (60 °C for 30 s), and finally, a dissociation cycle (melting curve; 60 to 95 °C, increasing 0.5 °C / 30 s) to verify that only one product was amplified. The primer concentrations were 10 pmol. Expressions of genes of interest were normalized by the expression of the housekeeping gene β-Actin and the relative quantification was calculated using the ΔCT formula. All samples for each experiment were run on the same plate in triplicate and the average values were calculated. Primers used in RT-qPCR are enlisted in Additional file 1: Table S2.

### Immunoblotting

Total proteins from HAEC in all experimental groups were extracted using native lysis buffer (Abcam) and cell debris was removed by centrifugation for 10 min at 12,000 × *g* at 4 °C. Immunoblotting was performed as previously reported [25]. Briefly, 30 µg of protein were subject to SDS-PAGE gel electrophoresis followed by transfer onto Immonobilon-P filter papers (Millipore) using the Trans-Blot Turbo Transfer System (Bio-Rad, Hercules, CA, USA). Membranes were blocked with 5% skimmed milk or 5% BSA in 0.1% Tween 20 (Sigma) in PBS for 1 h at room temperature (RT) and incubated overnight at 4 °C with specific primary antibodies against H3K27me3 (Millipore), H3 (Abcam), EZH2 (Abcam), UTX (Abcam), JMJD3 (Invitrogen), SOD1 (Abcam), SOD2 (Upstate Biotechnology), NOX4 (Santa Cruz Biotechnology) (1:1000), and GAPDH (Sigma) (1:10,000). Anti-rabbit and anti-mouse secondary antibodies were purchased from Santa Cruz Biotechnology and used at 1:10,000. The immunoreactive bands were detected by an enhanced chemiluminescence system (Millipore, USA). The intensity of the bands was quantified by densitometry using Image J v2.9.0 (NIH, Bethesda, MD, USA) and normalized by H3 (for H3K27me3) or GAPDH (for SOD1, SOD2, and NOX4) signals.

### Immunofluorescence studies

EZH2 and H3K27me3 were immunostained in HAEC and D-HAEC using specific antibodies. Briefly, cells grown on glass coverslips were fixed in 4% PFA for 10 min, permeabilizated with 0.1% Triton™ X-100 (Sigma) in D-PBS for 10 min, blocked with 1% BSA (Sigma) for 1 h at RT, and incubated with 1:100 primary anti-EZH2 (Thermo Fisher Scientific) and anti-H3K27me3 (Millipore) antibodies overnight at 4 ºC. After washing with PBS, 1:200 Alexa Fluor 594- and 488-conjugated antibodies (Abcam) were used as secondary antibodies for 2 h in darkness at RT, and nuclei were stained with Hoechst (2 µg/ml in PBS; Sigma) for 5 min. Aortic rings from WT and *db/db* mice were directly processed for immunohistochemistry or placed into 24-well plates containing EGM-2 culture media with GSK126 (5 µmol/l) or vehicle for 20 h. Then, they were fixed for 24 h in 4% PFA, hydrated in graded ethanol, embedded in paraffin and mounted on coated glass slides (SuperFrost Plus; Thermo Fisher Scientific). For antigen retrieval, slides were subjected to high-pressure boiling in citrate buffer (pH 6.0). After blocking with goat serum (Abcam), aortic cross-sections were incubated overnight (4 °C) with either of the following primary antibodies: rabbit polyclonal anti-H3K27me3 (Millipore), mouse monoclonal anti-EZH2 (Invitrogen), mouse monoclonals anti-SOD1 and anti-SOD2 (Santa Cruz Biotechnology) (1:100). A rabbit monoclonal antibody against the endothelial marker CD31 (Abcam; 1:100) was also used. Alexa Fluor 488 and 647-conjugated secondary antibodies (Abcam) were used (1:200) and nuclei were stained with Hoechst (2 µg/ml in PBS) for 20 min. Coverslips were mounted on slides with a fluorescence mounting medium (Agilent; Santa Clara, CA, USA).

### Confocal microscopy

Cells and tissue samples were visualized with a Leica TCS SP8 confocal laser scanning (Wetzlar, Germany) and a Nikon Eclipse Ti2 (Tokyo, Japan) microscope, respectively. Cells were examined with a 63X oil lens fitted with immersion oil (Leica; Wetzlar, Germany). 6–12 cells were randomly selected for each experimental condition and, depending on the cell depth, 5–8 stacks per channel were collected and projected in a single image. Aortic sections were visualized with 20X objective and 10 stacks per channel were collected. After the acquisition, image analysis was performed using ImageJ software.

### Measurement of superoxide anion

Superoxide anion (O_2_^−^) generation in HAEC and D-HAEC was assessed by electron spin resonance (ESR) spectroscopy analysis using the spin trap 1-hydroxy-3-methoxycarbonyl-2,2,5,5-tetramethyl-pyrrolidine (CMH) as described elsewhere [26]. Briefly, 6-well plated cells were washed with D-PBS and resuspended in 600 μL of Krebs-HEPES buffer supplemented with the following composition (µmol/l): deferoxamine methanesulfonate salt (DF; 25), diethyldithiocarbamic acid sodium salt (DETC; 5), and CMH (200) (Noxygen; Elzach, Germany). After 30 min incubation at 37 °C, cell suspensions were snap-frozen in liquid nitrogen and stored at − 80 °C. ESR spectra were recorded using a NOX-E.5-ESR spectrometer (Bruker; Billerica, MA, USA). Signals were quantified by measuring the total amplitude after correction of baseline and subtraction of background [26].

### NF-κB p65 binding activity assay

NF-κB p65 binding activity in HAEC and D-HAEC was measured by the TransAM™ NF-κB p65 activation protein assay kit (Active Motif; Carlsbad, CA, USA), following the manufacturer’s instructions. Briefly, 40 μg of whole cell lysate was added into a 96-well plate immobilized with consensus sequences for NF-κB p65 subunit (GGGACTTTCC) for 1 h at RT. Wells were washed with washing buffer following incubation with anti-NF-κB p65 antibody (Active Motif) for 1 h at RT. Horseradish peroxidase-conjugated secondary antibody was then added and the plate was incubated for an additional hour at RT. NF-κB p65 DNA binding was assessed by spectrophotometer at 450 nm on a VersaMax microplate reader (Ocean Springs, MI, USA).

### Monocyte adhesion assay

Monocyte adhesion to HAEC exposed to the different experimental conditions was tested in the presence and in the absence of TNF-α (5 mmol/l, 24 h). Briefly, human monocytes THP-1 were kindly donated by Dr. Magdalena Paolino (Karolinska Institute; Stockholm, Sweden) and grown in RPMI-1640 culture medium (Biowest; Nuaillé, France) supplemented with 10% FBS, 2 mol/l L-glutamine, 4,5 g/l glucose, and 1% penicillin/streptomycin, following the supplier’s recommendations. THP-1 cells were stained with 2 μmol/l of Calcein^AM^ fluorescent dye (Abcam) in serum-free media at 37 °C for 30 min, and washed with 10 mL of PBS. Stained cells were then resuspended in EBM2:RPMI-1640 medium (1:1), supplemented with 10% FBS, and co-cultured with HAEC under rotating conditions for 1 h at 37 °C. After incubation, non-adhering cells were removed with PBS and monolayers were fixed with 1% PFA. Co-cultures were observed under confocal microscopy and the number of adherent THP-1 cells was determined with ImageJ software.

### Experimental animal models

Animal care and protocols were approved by the regional ethical committee and conformed to the Guide for Care and Use of Laboratory Animals published by the US National Institutes of Health (NIH publication No. 85–23, revised 1996). Wild-type (WT) C57BL/6 male mice (6–8 weeks old) housed in the animal experimental core facility of Karolinska Institutet (Stockholm, Sweden) were sacrificed at the age of 12–18 weeks for isometric tension studies. Immunohistochemistry studies were performed in aortas from *db/db* and WT male mice (15–20 weeks old; Charles River Laboratories; Sulzfeld, Germany). All animals were kept in a 12:12-h light–dark cycle with free access to standard chow and water. Mice were anaesthetized with sodium pentobarbital (50 mg/kg i.p.) followed by thoracotomy. The entire aorta from the heart to the iliac bifurcation was excised and placed immediately in cold modified Krebs–Ringer bicarbonate solution (pH 7.4) of the following composition (mmol/l): NaCl (118.6), KCl (4.7), CaCl2 (2.5), KH2PO4 (1.2), MgSO4 (1.2), NaHCO3 (25.1), glucose (11.1), and calcium EDTA (0.026) (Sigma). The aortas were cleaned from adhering fat and connective tissues under a dissection microscope and subsequently cut transversely into 2 mm rings.

### Isometric tension studies

Aortic rings from C57BL/6 mice were placed into 24-well plates containing EGM-2 culture media with either normal (5 mmol/l) or high glucose concentration (25 mmol/l) in the absence and in the presence of GSK126 (5 µmol/l) for 20 h. Then, the aortic rings were mounted in an isometric force transducer (multi-Myograph 610 M; Danish Myo Technology, Hinnerup, Denmark), suspended in an organ chamber filled with 6 mL Krebs–Ringer bicarbonate solution at 37 °C, and bubbled with 95% O_2_ and 5% CO_2_. The internal diameter was set at a tension equivalent to 0.9 times the estimated diameter at 100 mmHg. Resting tension was gradually increased to 2 mN. Following a 30 min equilibration period, aortic rings were exposed to potassium chloride twice (50 mmol/l, Sigma). After pre-contraction with phenylephrine (10^−6^ mol/l; Sigma), endothelium-dependent and independent relaxations were assessed by acetylcholine (Ach; 10^−9^–10^−4^ mol/l; Sigma) and nitroprusside (SNP; 10^−10^–10^−5^ mol/l, Sigma), respectively. Incubation of aortas with a high concentration of mannitol (25 mmol/l) did not affect vascular responses to Ach and SNP (Additional file 1: Fig. S2). Isometric forces for the different data points were quantified with LabChart™ software, and relaxations were expressed as a percentage of the pre-contraction plateau tension. Several aortic rings from the same animal were studied in parallel.

### Statistical analysis

All data are presented as mean ± standard error of the mean (SEM), and statistical analysis was performed using GraphPad Prism Software (version 9.0.1). The normality distribution of the samples was assessed by the Shapiro–Wilk normality test. Unpaired t for parametric and Mann–Whitney tests for non-parametric data were used to determine the significance between two groups. One-way ANOVA with Tukey’s multiple comparisons and Kruskal–Wallis with Dunn’s multiple comparison tests were used for comparison among several groups of parametric and non-parametric data, respectively. Two-way ANOVA was used for the myograph studies. A p-value less than 0.05 was considered statistically significant.

## Results

### High glucose induces H3K27me3 via derangement of histone-modifying enzymes

**Fig. 1:**
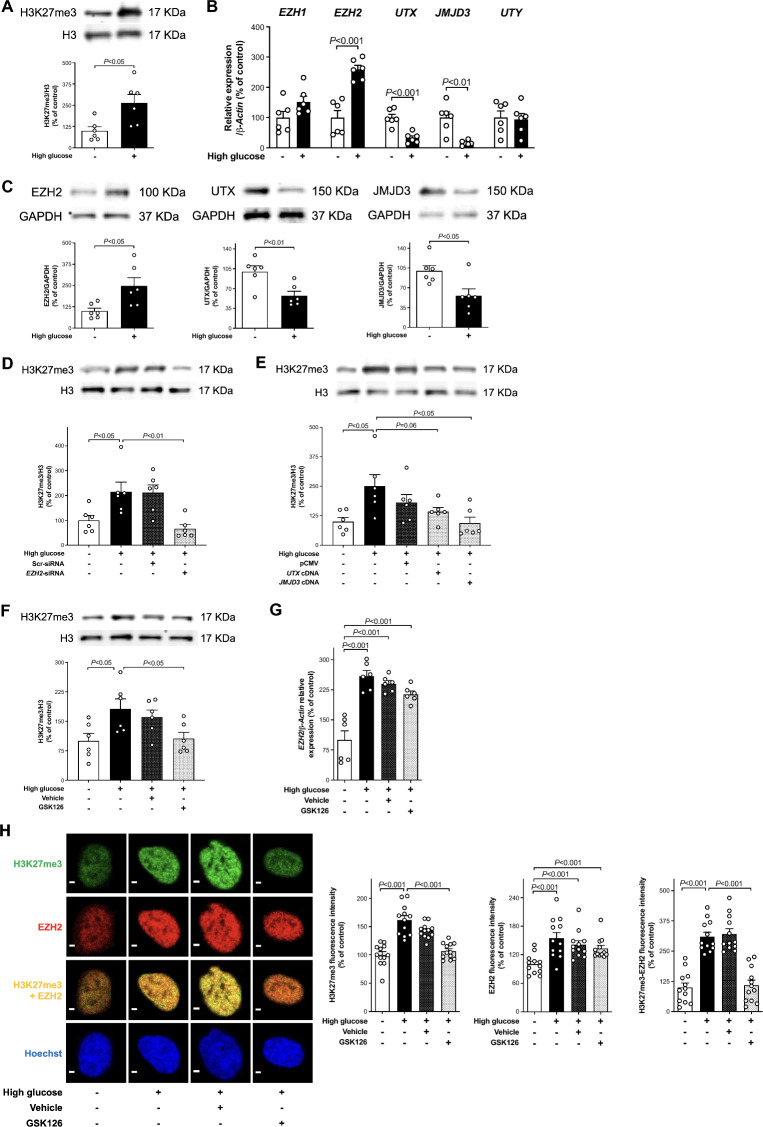
High glucose triggers repressive H3K27me3 via derangement of histone-modifying enzymes. **A** Representative western blot images and relative densitometric quantifications showing H3K27me3 expression in HAEC exposed to normal (5 mmol/l) and high (25 mmol/l) glucose (n = 6/group). **B** Histone methyltransferases (*EZH1*, *EZH2*) and demethylases (*UTX*, *JMJD3*, *UTY*) mRNAs in the two experimental groups (n = 6/group) assessed by RT-qPCR. **C** Representative western blot images and densitometric quantifications of EZH2, UTX, and JMJD3 expression in HAEC exposed to normal and high glucose (n = 6/group). **D**, **E** Representative western blot images and densitometric quantifications showing H3K27me3 expression after reprogramming of chromatin-modifying enzymes (n = 6/group). **F** H3K27me3 protein expression in the presence of EZH2 selective inhibitor GSK126 (5 µmol/l) or vehicle alone (n = 6/group). **G** *EZH2* gene expression assessed by RT-qPCR (n = 6/group) in HAEC exposed to the same experimental conditions. **H** Confocal microscopy images of H3K27me3 (green), EZH2 (red), and EZH2/H3K27me3 colocalization (yellow), and relative quantification of fluorescence intensity. Cell nuclei are stained with Hoechst (blue). Scale bar = 2 μm. (n = 12/group)

HAEC were exposed to normal (5 mmol/l) or high (25 mmol/l) concentrations of glucose for 20 h and immunoblotting studies showed an increase of the repressive epigenetic mark H3K27me3 in high glucose-treated cells (Fig. 1A). The exposure time was chosen after testing different time points (Additional file 1: Fig. S3). A PCR array was performed to investigate the histone-modifying enzymes involved in such H3K27me3 change. Upregulation of H3K27 methyl writing *EZH2* and reduced expression of demethylases *UTX* and *JMJD3* genes were observed in cells exposed to high glucose (Fig. 1B). By contrast, methyltransferase *EZH1* and demethylase *UTY* gene expression was not significantly changed (Fig. 1B). Protein levels of EZH2, UTX, and JMJD3 were altered according to the gene expression (Fig. 1C).

Consistently, editing of deranged H3K27-chromatin modifiers by small interfering EZH2 RNA (siRNA) and UTX or JMJD3 overexpressing vectors was able to blunt high glucose-induced H3K27me3 increase (Fig. 1D and E, and Additional file 1: Fig. S4).

### Pharmacological inhibition of EZH2 restores H3K27me3 levels

The EZH2 selective inhibitor GSK126 (5 µmol/l) blunted high glucose-induced H3K27me3 signature (Fig. 1F) without exerting any effect on the upregulation of *EZH2* gene (Fig. 1G). These results were confirmed by confocal microscopy showing that the nuclei of HAEC exposed to high glucose exhibited a markedly enhanced specific immunostaining with antibodies against histone methyltransferase EZH2 and H3K27me3 compared to control cells (Fig. 1H). Accordingly, inhibition of EZH2 with GSK126 abolished the increase of the H3K27me3 signal without exerting any significant effect on EZH2 expression (Fig. 1H). To determine whether EZH2 and H3K27me3 colocalize in HAEC exposed to high glucose, a double staining approach was adopted. These cells showed the colocalization of green and red spots that is represented in yellow in the merged image (Fig. 1H). Treatment with GSK126 prevented such colocalization by inhibiting EZH2-induced H3K27me3 (Fig. 1H).

### EZH2-dependent H3K27me3 promotes oxidative stress

**Fig. 2:**
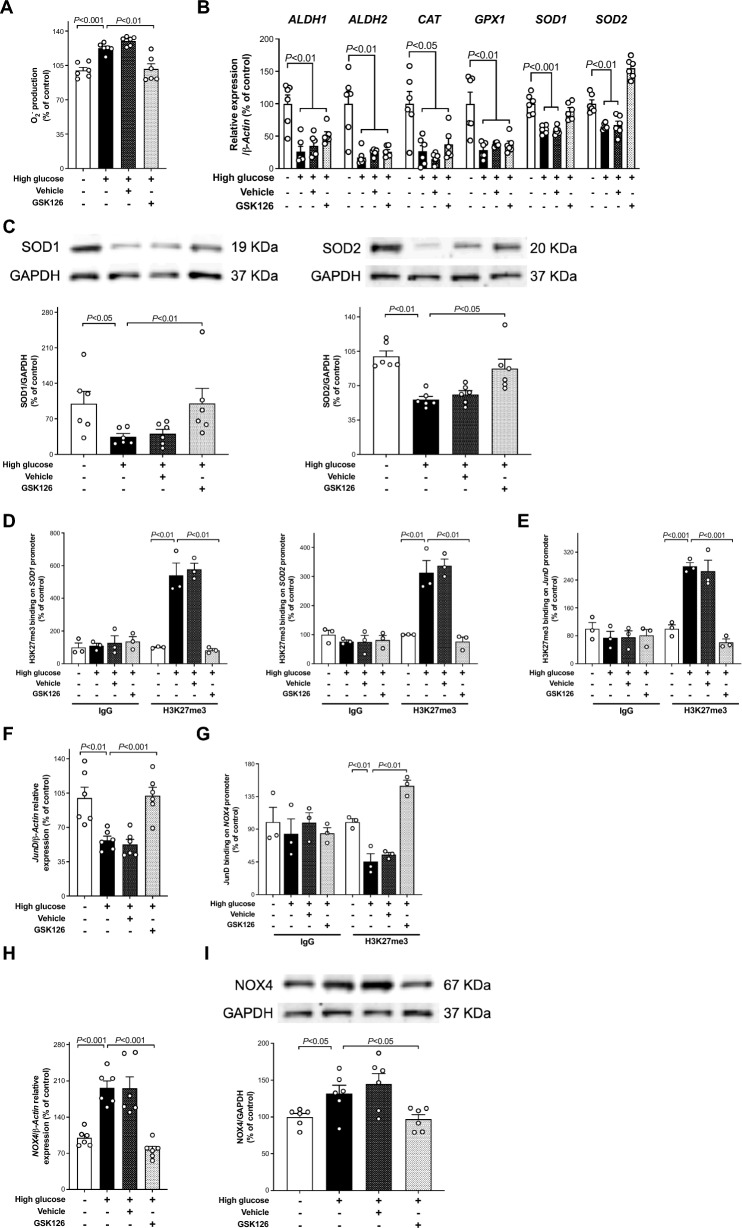
EZH2-mediated H3K27me3 signature contributes to oxidative stress. **A** Electron spin resonance (ESR) spectroscopy analysis of O_2_^−^ production, **B** expression of ROS scavenging enzymes *ALDH1*, *ALDH2*, *CAT*, *GPX1*, *SOD1* and *SOD2* genes and **C** representative Western blots images and densitometric quantifications of SOD1 and SOD2 proteins in HAEC exposed to normal (5 mmol/l) and high (25 mmol/l) glucose treated with EZH2 inhibitor GSK126 (5 µmol/l) or vehicle alone (n = 6/group). **D** ChIP-qPCR assay showing the binding of H3K27me3 to *SOD1* and *SOD2* promoters in high glucose-treated cells and the inhibitory effect exterted by GSK126 (5 µmol/l; n = 3/group). **E** The interaction of H3K27me3 with *JunD* promoter in HAEC exposed to high glucose was also abolished by EZH2 inhibitor GSK126 as shown by ChIP-qPCR assay (n = 3/group), **F–I** Downregulation of *JunD* mRNA (n = 6/group), **G** JunD binding on *NOX4* promoter (n = 3/group), and subsequent upregulation of **H** NOX4 gene and **I** protein expression (n = 6/group) in HAEC exposed to high glucose were blunted by GSK126 (5 µmol/l) but not DMSO vehicle alone. IgG controls of ChIP-qPCR assay are also shown

EZH2-induced histone modification drives glucose-mediated increase of superoxide anion (O_2_^−^) generation in HAEC as assessed by electron spin resonance (ESR) spectroscopy (Fig. 2A). Indeed, methyltransferase inhibition with GSK126 restored control levels of O_2_^−^ (Fig. 2A).

### H3K27me3 signature and ROS-scavenging enzyme expression

An RT-qPCR array was performed to assess the role of the repressive epigenetic mark H3K27me3 on the expression of key antioxidant genes. Gene expression of ROS-scavenging enzymes such as *ALDH1*, *ALDH2*, *CAT*, *GPX1*, *SOD1*, and *SOD2* was downregulated in cells exposed to high glucose compared to control conditions (Fig. 2B). However, only the expression of *SOD1* and *SOD2* mRNA was significantly restored in the presence of methyltransferase inhibitor GSK126 (Fig. 2B). The rescuing effect of EZH2 inhibitor was also confirmed by measuring SOD1 and SOD2 protein levels (Fig. 2C). To assess the epigenetic regulation of *SOD1* and *SOD2* genes, chromatin immunoprecipitation (ChIP-qPCR) assay was performed. We found that repressive H3K27me3 binding to *SOD1* and *SOD2* promoters was enhanced in HAEC exposed to high glucose (Fig. 2D). Inhibition of EZH2 with GSK126 abolished H3K27me3 on *SOD1* and *SOD2* promoters (Fig. 2D). Interestingly, EZH2 siRNA, and UTX or JMJD3 overexpressing vectors (Additional file 1: Fig. S4A and B) confirmed the contribution of high glucose-induced repressive epigenetic mark H3K27me3 to O_2_^−^ generation via downregulation of *SOD1* and *SOD2* genes (Additional file 1: Fig. S5A-D).

### EZH2-induced H3K27me3, AP-1 transcription factor JunD, and NOX4 expression

Since AP-1 transcription factor JunD has been shown to protect endothelial cells from oxidative stress by regulating the expression of NADPH oxidase subunit NOX4, we investigated the role of EZH2-mediated epigenetic regulation of JunD on NOX4 expression. Our ChIP-qPCR experiments showed a significant increase of H3K27me3 binding to *JunD* promoter (Fig. 2E). The subsequent downregulation of *JunD* mRNA (Fig. 2F) reduced the interaction between JunD and *NOX4* promoter (Fig. 2G), leading to increased gene and protein expression of NOX4 (Fig. 2H and I). Consistently, treatments with GSK126, EZH2 siRNA, and UTX or JMJD3 overexpressing vectors were able to inhibit EZH2-induced, H3K27me3-dependent JunD downregulation and the subsequent increase of *NOX4* gene and protein expressions (Fig. 2E–I, and Additional file 1: Fig. S5E and F).

### NF-κB-dependent inflammatory pathway

**Fig. 3:**
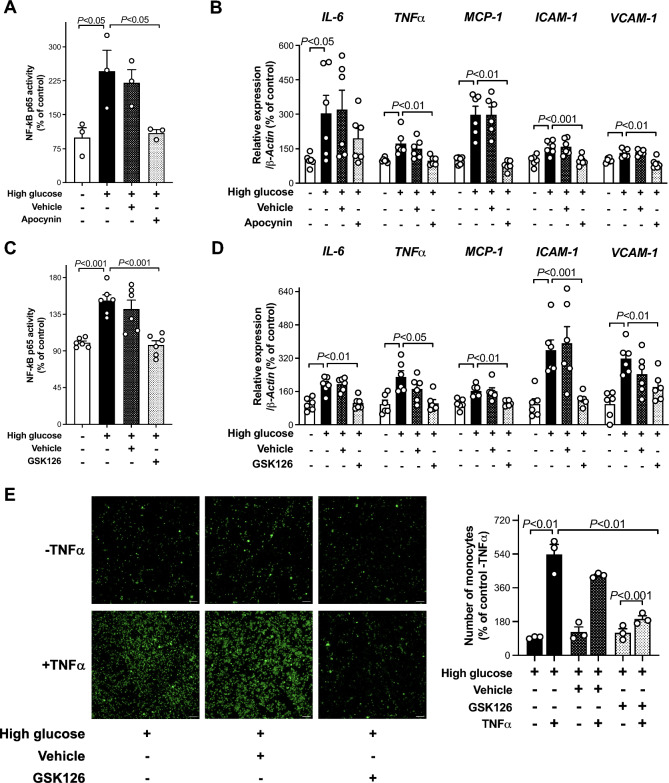
EZH2-mediated H3K27me3 signature contributes to NF-*κ*B p65-dependent inflammatory changes. **A**, **C** Effect of NADPH oxidase inhibitor apocynin (100 µmol/l) and GSK126 (5 µmol/l) on high glucose-induced increase of NF-κB p65 binding activity (n = 3–6/group). **B**, **D** RT-qPCR showing gene expression of inflammatory markers (n = 6/group) in HAECs exposed to high glucose alone in the presence of apocynin (100 µmol/l), GSK126 (5 µmol/l), or vehicle alone. **E** Representative images and relative quantification of monocyte adhesion to HAEC exposed to high glucose in the presence and in the absence of TNFa (5 mmol/l) and treated with GSK126 (5 µmol/l) or vehicle alone (n = 3/group). Scale bar = 100 μm. *IL-6* interleukin-6, *TNFα* tumor necrosis factor α, *MCP-1* monocyte chemoattractant factor-1, *ICAM-1* intercellular adhesion molecule 1, *VCAM-1* vascular cell adhesion molecule 1

The contribution of EZH2-induced H3K27me3 mark to oxidative stress dependent inflammatory changes in HAEC exposed to high glucose was determined by assessing the effect of NADPH oxidase inhibitor apocynin and GSK126 on NF-κB p65 binding activity as well as cytokine and adhesion molecule gene expression. Interestingly enough, not only apocynin but also EZH2 inhibitor blunted NF-κB p65 binding activity and rescued control expression of inflammatory genes (Fig. 3A–D). Such rescuing effect was also observed in the presence of EZH2 siRNA and UTX or JMJD3 overexpressing vectors (Additional file 1: Fig. S6A and B). To test the functional significance of the observed expression derangements we determined monocyte adhesion to HAEC. TNFa-induced monocyte adhesion in HAEC exposed to high glucose was significantly blunted by GSK126 (Fig. 3E).

### GSK126 restores glucose-induced endothelial dysfunction

**Fig. 4:**
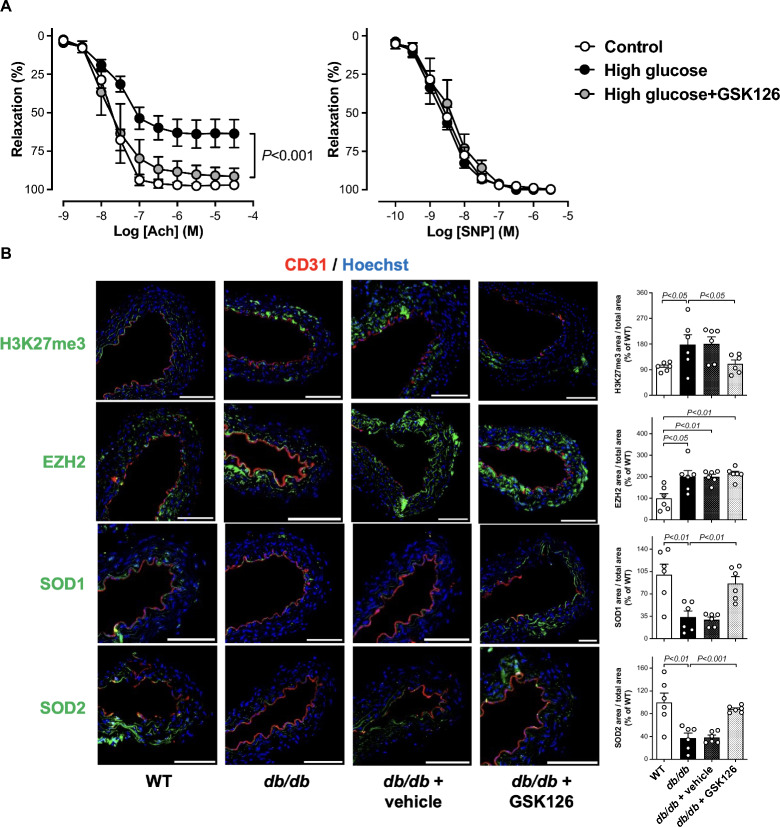
GSK126 recovers high glucose-induced endothelial dysfunction. **A** Endothelium-dependent relaxations to acetylcholine (Ach) and endothelium-independent relaxations to sodium nitroprusside (SNP) in mouse aortic rings after exposure to normal (5 mmol/l) and high (25 mmol/l) glucose in the presence and in the absence of GSK126 (5 µmol/l; n = 4–6/group). **B** Representative confocal images and quantification of H3K27me3, EZH2, SOD1, and SOD2 protein expression in aortas from WT, *db/db* mice, and *db/db* mice exposed to vehicle or GSK126 (5 µmol/l; n = 6/group). Scale bar = 500 μm

We next investigated whether the EZH2-H3K27me3-driven oxidative phenotype contributes to high glucose-induced endothelial dysfunction. Aortic rings from wild-type mice were incubated for 20 h in a culture medium with normal (5 mmol/l) or high (25 mmol/l) concentration of glucose in the presence and in the absence of GSK126 (5 µmol/l). Of note, isometric tension studies showed that treatment with GSK126 restored endothelium-dependent relaxations to acetylcholine in aortic rings exposed to high glucose (Fig. 4A). As expected, the endothelium-independent relaxations to SNP did not differ between experimental groups (Fig. 4A).

### EZH2-H3K27me3 epigenetic axis and diabetes

**Fig. 5:**
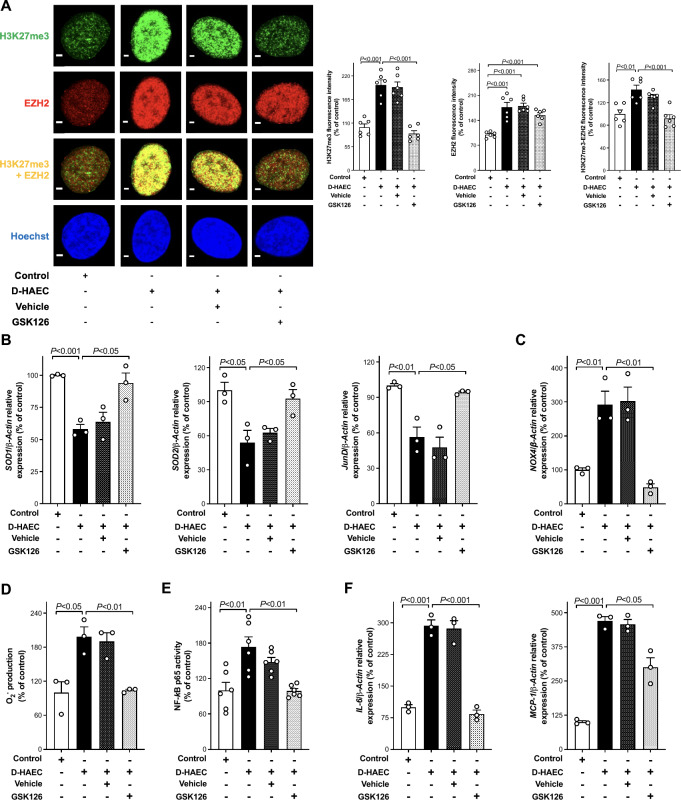
Targeting EZH2-H3K27me3 epigenetic signature rescues abnormal phenotypes in endothelial cells isolated from patients with diabetes. **A** Representative confocal microscopy images of H3K27me3 (green), EZH2 (red), and EZH2/H3K27me3 colocalization (yellow) and densitometric quantification of fluorescence intensity. Cell nuclei are stained with Hoechst (blue). Scale bar = 2 μm. n = 6/group. **B** and **C** RT-qPCR arrays showing gene expression of *SOD1*, *SOD2*, *JunD*, and **C** *NOX4*, respectively (n = 3/group). **D** electron spin resonance (ESR) spectroscopy analysis of O_2_^−^ production (n = 3/group). **E** NF-κB p65 binding activity (n = 6/group), and **F** gene expression of *IL-6* and *MCP-1* (n = 3/group). IL-6, interleukin-6; MCP-1, monocyte chemoattractant factor-1

To determine whether the EZH2-H3K27me3 epigenetic axis is also active in the setting of diabetes, additional experiments were performed in aortas from *db/db* mice and in endothelial cells isolated from patients with diabetes (D-HAEC). Consistently, increased protein levels of EZH2 and H3K27me3 protein levels were mirrored by a decreased expression of SOD1 and SOD2 in the endothelial cells of aortas isolated from *db/db* as compared to WT mice (Fig. 4B). An increase in EZH2 and H3K27me3 fluorescence was also confirmed in D-HAEC by confocal microscopy (Fig. 5A). GSK126 (5 µmol/l) abolished such increased immunostaining and colocalization (Figs. 4B and 5A). Interestingly enough, the inhibitory effect of GSK126, on one hand restored control O_2_^−^ production by modulating *SOD1*, *SOD2*, *JunD and NOX4* gene expression, and also blunted inflammatory pathways by bringing NF-κB p65 binding activity as well as *MCP-1* and *IL-6* mRNAs to control levels (Fig. 5B–F).

## Discussion

The present study demonstrates that inhibition of EZH2-driven H3K27me3 signature blunts glucose-induced adverse transcriptional programs in the endothelium. Several lines of evidence support our conclusion: (i) HAEC exposed to high glucose exhibit mRNA and protein upregulation of H3K27 methyl-writing enzyme EZH2 as well as reduced expression of demethylases UTX and JMJD3 with concomitant increase of the repressive epigenetic mark H3K27me3; (ii) binding of H3K27me3 to the promoter region of *SOD1*, *SOD2*, and *JunD* genes is responsible for their reduced transcription; (iii) loss of JunD transcriptional inhibition is associated with increased expression of NADPH oxidase subunit NOX4 and O_2_^−^ generation; (iv) H3K27me3-driven oxidative stress contributes to increase NF-κB p65 binding activity and inflammation; (v) reduction of H3K27me3 levels either by EZH2 selective inhibitor GSK126 or reprogramming of H3K27-chromatin modifiers via EZH2 siRNA and UTX or JMJD3 overexpressing vectors rescue all these events; (vi) last, but not least, EZH2 and H3K27me3 are markedly enhanced in *db/db* mice aorta as well as in endothelial cells obtained from patients with diabetes and correlate with oxidative stress and inflammation, suggesting a potential translation of our findings to the disease context.

Over the last 30 years, the detrimental effects of hyperglycemia on the vessel wall have promoted major research efforts leading to the understanding that high glucose decreases the availability of endothelium-derived nitric oxide and triggers inflammation via an array of mechanisms involving overproduction of reactive oxygen species (ROS) [27]. However, the precise molecular mechanisms by which high glucose leads to oxidative and inflammatory endothelial phenotypes remain poorly elucidated. Although the enzymatic activity of histone methyltransferases writes covalent modifications on histone core proteins to control gene transcription [28], few studies have investigated the effect of histone methylation on ROS generation in endothelial cells exposed to high glucose [29]. H3K27me3 signature is catalyzed by PRC2 complex containing either EZH1 (KMT6B) or EZH2 (KMT6) as catalytic subunits. H3K27me3 is associated with transcriptional repression [30]. On the other hand, demethylation of H3K27 is principally carried out by UTX (KDM6A), JMJD3 (KDM6B), and UTY (KDM6C) demethylases and results in the activation of gene expression [31–34]. Our findings clearly show that high glucose-induced H3K27me3 is due to an imbalance in the expression of histone methyltransferase EZH2 and demethylases UTX and JMJD3 genes. Even if we did not find changes in methyltransferase EZH1 or demethylase UTY expression, we cannot completely rule out their involvement. As a matter of fact, the inhibition of H3K27me3 obtained by both EZH2 siRNA and UTX or JMJD3 overexpressing vectors indicates dynamic cooperativity of methyl-writers and methyl-erasers in triggering H3K27me3 and, hence, inhibition of downstream target gene expression [35]. Interestingly, our results align closely with prior findings by Brasacchio et al. [36], which highlighted the interplay of other histone methylase and demethylase enzymes driving H3K4 methylation as well as H3K9 demethylation in similar experimental conditions. Understanding the epigenome alterations in the setting of hyperglycemia is of paramount importance to establish novel therapeutic options to mitigate its cardiovascular complications.

H3K27me3 signature is associated with transcriptional repression, and it is regarded as a critical methylation checkpoint during tissue development and stem cell fate determination [37]. Moreover, EZH2-induced H3K27me3 works as a master regulator of multiple cellular processes, including cell cycle, autophagy, apoptosis, DNA damage, and cellular senescence [38]. The development of small molecules that effectively inhibit EZH2 enzymatic activity has yielded some promising results. Clinical trials have reported on EZH2 inhibitors’ safety and efficacy in cancer [39], but their putative use in cardiometabolic disease has not been explored [40]. GSK126 is a highly selective inhibitor of EZH2 methyltransferase activity (> 1000-fold selective for EZH2 as compared to other human methyltransferases) [41].

Here, we demonstrate that in HAEC exposed to high glucose editing of deranged EZH2 expression by small interfering EZH2 siRNA rescued adverse H3K27me3 signature. The selective pharmacological inhibition of EZH2 with GSK126, blunting H3K27me3 without any effect on EZH2 expression [42], was able to inhibit glucose-induced oxidative stress and inflammatory phenotypes. Increased EZH2 expression and activity have been reported in high glucose-stimulated retinal endothelial cells and are linked to dysregulation of miR-200b and diabetic retinopathy [43]. By contrast, an earlier study showed that EZH2 is downregulated in HUVECs from patients with gestational diabetes [44]. Other studies have demonstrated that EZH2 is involved in cardiac hypertrophy, ROS generation, and inflammation [29, 45]. Increased EZH2 activity inhibits SOD1 expression leading to ROS accumulation that contributes to the progression of pulmonary artery hypertension [29]. It was also reported that targeting EZH2 in erythroid cells protects against oxidative stress [46]. ROS-scavenging enzyme SOD is a major cellular antioxidant defence system [47]. Thus, SOD plays a critical role in protecting NO from inactivation and, hence, endothelial dysfunction [48]. In the present study, we demonstrate that endothelial O_2_^−^ generation is associated with H3K27me3-induced decrease of SOD1 and SOD2 expression. Indeed, our ChIP-qPCR experiments revealed an increase of H3K27me3 binding to the promoter region of *SOD1* and *SOD2* genes. Treatment with EZH2 methyltransferase inhibitor GSK126 preserved active transcription of SOD1 and SOD2 and, hence, blunted O_2_.^−^ production in the presence of high glucose. By contrast, our results show that EZH2-mediated H3K27me3 is not involved in the downregulation of other antioxidant genes such as *ALDH1*, *ALDH2*, *CAT*, and *GPX1* because treatment with GSK126 was not able to rescue their expression. Although these observations highlight the specificity of the H3K27me3 signature for SOD1 and SOD2 genes, more investigation is needed to elucidate the whole pattern of H3K27me3 target genes in endothelial cells. For instance, it has been recently observed that transmembrane protein 215 (TMEM215) is a novel target gene of EZH2-mediated transcriptional repression. [49]

The results presented here also suggest that glucose-induced O_2_^−^ production is associated with transcriptional derangement of other redox gene expression. NADPH oxidase subunit NOX4 is a primary source of ROS and contributes to endothelial dysfunction in the setting of diabetes [50, 51]. Indeed, targeting NOX4 attenuates ROS generation and restores endothelial homeostasis [50]. Our ChIP-qPCR analysis shows that a reduced binding of AP-1 transcription factor JunD to the promoter region of NOX4 gene upregulates NOX4 expression. Interestingly enough, glucose-induced downregulation of transcription factor JunD is mediated by EZH2. Indeed, treatment with GSK126 removes the EZH2 methyl-writing repressive tag on JunD promoter and rescues its inhibitory effect on NOX4 expression. Our findings are in line with the role of JunD as a major gatekeeper against oxidative stress [52, 53]. We have recently reported that JunD downregulation in cardiomyocytes alters the balance between oxidant and antioxidant enzymes leading to oxidative stress, inflammation, and myocardial dysfunction in experimental and human diabetes [54]. Accordingly, EZH2 silencing has been found to improve cardiac function and prevent cardiomyocyte apoptosis in experimental diabetes [55]. EZH2 inhibition by GSK126 protects against glucolipotoxicity-induced endoplasmic reticulum stress and apoptosis in β-cells [56]. Moreover, GSK126 was shown to be able to restore β-cells transcriptional program even in the presence of significant β-cell destruction such as in type 1 diabetes [57]. Furthermore, inhibition of the EZH2/p38 signalling pathway has been shown to decrease apoptosis and inflammation, and improve renal function in acute kidney injury induced by ischemia–reperfusion [58]. These reports collectively provide compelling evidence for the translational importance of inhibiting EZH2 and are in line with our results proving the ability of GSK126 to protect against hyperglycemia-induced endothelial dysfunction.

It is also well-established that hyperglycemia-induced oxidative stress in the endothelium triggers inflammatory changes and both of them contribute to atherosclerotic vascular complications in this setting. [3]. In accordance with the notion that a deranged redox balance may favor inflammation via activation of NF-kB p65 pathway, in our experimental setting both GSK126 as well as NADPH oxidase inhibitor apocynin suppressed NF-kB p65 binding activity and gene expression of inflammatory mediators (*IL-6*, *TNFα*, *MCP1*) as well as adhesion molecules (*ICAM-1*, *VCAM-1*). Indeed, GSK126 has been recently shown to decrease the expression of inflammatory and proatherogenic genes in macrophages and atherosclerotic plaques, attenuating the development of atherosclerosis in ApoE^−/−^ mice fed with high-fat diet [42]. Taking into consideration that H3K27me3-rich genomic regions function as main silencers of gene expression [9], we cannot rule out the involvement of other inflammatory genes in the glucose-induced derangements of endothelial phenotype. It was recently reported that H3K27me3 leads to inflammation also by transcription factor KLF2 in human umbilical vein endothelial cells exposed to an intermittent high glucose environment [59]. In contrast with our findings, this study shows a decreased phosphorylation of EZH2 leading to its nuclear translocation and, apparently, no change in EZH2 total protein expression after high glucose treatment for 72 h. In this regard, it is important to consider that different endothelial cells and experimental settings might explain this discrepancy. In addition to our current results on EZH2-induced H3K27me3, previous studies have demonstrated that high glucose-induced upregulation of NF-*k*B p65 gene is also associated with concomitant upregulation of methyltransferase SET7 and demethylase LSD1 driving H3K4me1 activation and H3K9me2/3 supression, respectively [36, 60].

Oxidative stress and inflammation have been closely linked to the pathophysiological processes implicated in diabetes-induced endothelial dysfunction. In this regard, it is noteworthy that our isometric tension studies performed in the aorta of wild-type mice exposed to high glucose also demonstrate that treatment with GSK126 protects against glucose-induced impairment of endothelium-dependent relaxations to acetylcholine. Another important result of the present study is that the H3K27me3 epigenetic signature drives phenotype derangements in the aorta of *db/db* mice and in endothelial cells isolated from patients with diabetes (D-HAEC). These additional findings pave the way for pilot preclinical studies targeting EZH2-mediated H3K27me3 signature with GSK126 in the setting of diabetes.

## Conclusion

Our work unmasks H3K27me3 as a detrimental epigenetic mediator promoting oxidative stress and inflammation in the endothelium. In addition, the present findings provide new insights to broaden the therapeutic use of EZH2 selective inhibitor GSK126, which has been, so far, only investigated as an anticancer drug [61]. Such a novel target against glucose-induced adverse transcriptional programs might represent the epigenetic way to cardiovascular protection in the setting of diabetes.

### Supplementary Information

**Additional file 1: Fig. S1.** HAEC viability. Cells were exposed to increasing concentrations of GSK126 (0-15 μmol/l) for 20 h (n=3/group). **Fig. S2.** Mannitol and endothelial function. (A) Endothelium-dependent relaxations to acetylcholine (Ach) and (B) endothelium-independent relaxations to sodium nitroprusside (SNP) after 20-hour exposure to normal (5 mmol/l) or high (25 mmol/l) concentrations of mannitol (n=4-6/group). **Fig. S3.** Time-course of high glucose concentration and H3K27me3 expression. Representative western blot images and relative densitometric quantifications showing H3K27me3 protein in HAEC exposed to high glucose (25 mmol/l). The open bar represents H3K27me3 in cell exposed to normal glucose (5 mmol/l, n=3/group). **Fig. S4.** Reprogramming of chromatin modifying enzymes by EZH2 siRNA, and UTX or JMJD3 overexpressing vectors. (A) mRNA and (B) protein expression of EZH2, UTX, and JMJD3 in HAEC exposed to normal (5 mmol/l) and high glucose (25 mmol/l). Scramble-siRNA and pCMV were used as controls for siRNA-mediated knockdown and vector-based overexpression, respectively (n=3-6/group). **Fig. S5.** Reprogramming of EZH2, UTX and JMJD3 expression levels blunts glucose-induced oxidative stress. **Fig. S6.** Reprogramming of EZH2, UTX and JMJD3 expression abolishes glucose induced inflammation. RT-qPCR showing IL-6 and MCP-1 gene expression in HAEC exposed to normal (5 mmol/l) or high glucose (25 mmol/l) in the presence and in the absence of (A) EZH2 siRNA, and (B) UTX and JMJD3 overexpressing vectors. Scramble-siRNA and pCMV were used as controls for siRNA-mediated knockdown and vector-based overexpression, respectively (n=6/group). **Table S1.** Primers used in RT-qPCR experiments. **Table S2.** Primers used in ChIP-qPCR assays.

## Data Availability

The data that support the findings of this study can be shared by the corresponding author upon reasonable request.

## Abbreviations

ChIP-qPCR: Chromatin immunoprecipitation couple to qPCR
CMH: 1-Hydroxy-3-methoxycarbonyl-2,2,5,5-tetramethyl-pyrrolidine
D-HAEC: Human aortic endothelial cells isolated from individuals with diabetes
ESR: Electron spin resonance
GSK126: GSK-2816126
H3K27me3: Trimethylation of histone H3 lysine 27
HAEC: Human aortic endothelial cells
O_2_^−^: Superoxide anion
PRC2: Polycomb repressive complex 2
ROS: Reactive oxygen species
