# Supplementary material for: Repressive H3K27me3 drives hyperglycemia-induced oxidative and inflammatory transcriptional programs in human endothelium
Source: Cardiovasc Diabetol. 2024 Apr 5;23:122. doi: 10.1186/s12933-024-02196-0 (PMC10998410; doi:10.1186/s12933-024-02196-0)
Supplement: Supplementary file 1 — Additional file 1: Fig. S1. HAEC viability. Cells were exposed to increasing concentrations of GSK126 (0-15 μmol/l) for 20 h (n=3/group). Fig. S2. Mannitol and endothelial function. (A) Endothelium-dependent relaxations to acetylcholine (Ach) and (B) endothelium-independent relaxations to sodium nitroprusside (SNP) after 20-hour exposure to normal (5 mmol/l) or high (25 mmol/l) concentrations of mannitol (n=4-6/group). Fig. S3. Time-course of high glucose concentration and H3K27me3 expression. Representative western blot images and relative densitometric quantifications showing H3K27me3 protein in HAEC exposed to high glucose (25 mmol/l). The open bar represents H3K27me3 in cell exposed to normal glucose (5 mmol/l, n=3/group). Fig. S4. Reprogramming of chromatin modifying enzymes by EZH2 siRNA, and UTX or JMJD3 overexpressing vectors. (A) mRNA and (B) protein expression of EZH2, UTX, and JMJD3 in HAEC exposed to normal (5 mmol/l) and high glucose (25 mmol/l). Scramble-siRNA and pCMV were used as controls for siRNA-mediated knockdown and vector-based overexpression, respectively (n=3-6/group). Fig. S5. Reprogramming of EZH2, UTX and JMJD3 expression levels blunts glucose-induced oxidative stress. Fig. S6. Reprogramming of EZH2, UTX and JMJD3 expression abolishes glucose induced inflammation. RT-qPCR showing IL-6 and MCP-1 gene expression in HAEC exposed to normal (5 mmol/l) or high glucose (25 mmol/l) in the presence and in the absence of (A) EZH2 siRNA, and (B) UTX and JMJD3 overexpressing vectors. Scramble-siRNA and pCMV were used as controls for siRNA-mediated knockdown and vector-based overexpression, respectively (n=6/group). Table S1. Primers used in RT-qPCR experiments. Table S2. Primers used in ChIP-qPCR assays. [file 12933_2024_2196_MOESM1_ESM.pdf]

ADDITIONAL FILES

Repressive H3K27me3 drives hyperglycemia-induced oxidative and inflammatory transcriptional programs in human endothelium

Julia Sánchez-Ceinos[1†], Shafaat Hussain[1,2†], Abdul Waheed Khan[1,3], Liang Zhang[1], Wael Almahmeed[4], John Pernow[1], Francesco Cosentino[1*]

[1]Cardiology Unit, Department of Medicine-Solna, Karolinska Institutet, Karolinska University Hospital; Stockholm, Sweden. [2]Department of Molecular and Clinical Medicine, University of Gothenburg; Gothenburg, Sweden. [3]Department of Diabetes, Central Clinical School, Monash University; Melbourne, Australia. [4]Heart and Vascular Institute, Cleveland Clinic Abu Dhabi, Abu Dhabi, UAE.

[†]Julia Sánchez-Ceinos and Shafaat Hussain have contributed equally to this work.

***Address for correspondence:**

Francesco Cosentino, MD, PhD

Cardiology Unit

Department of Medicine-Solna

Karolinska Institutet, Karolinska University Hospital

171 76, Stockholm, Sweden

Email: francesco.cosentino@ki.se

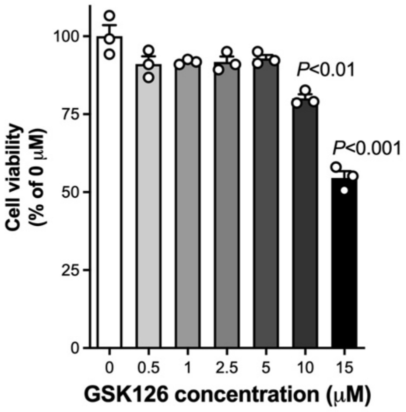

Fig. S1 HAEC viability. Cells were exposed to increasing concentrations of GSK126 (0-15 μmol/l) for 20 h (n=3/group).

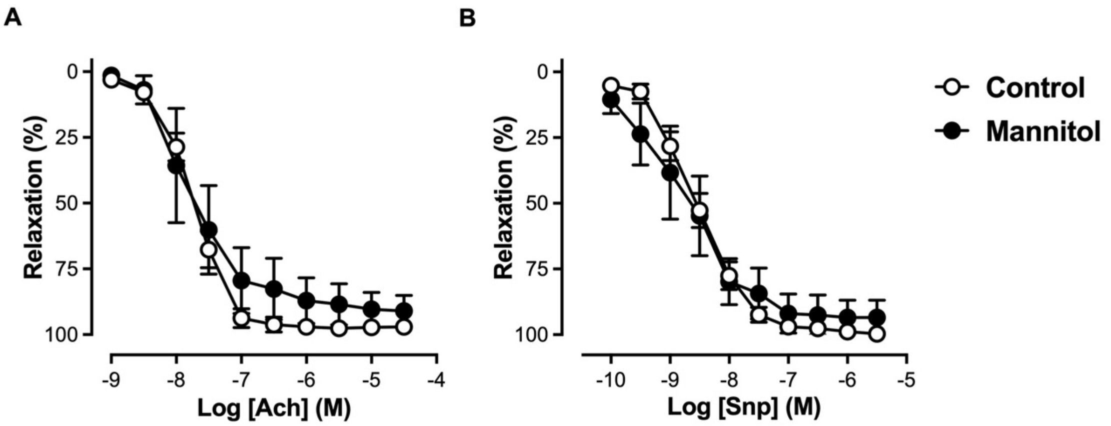

Fig. S2 Mannitol and endothelial function. (A) Endothelium-dependent relaxations to acetylcholine (Ach) and **(B)** endothelium-independent relaxations to sodium nitroprusside (SNP) after 20-hour exposure to normal (5 mmol/l) or high (25 mmol/l) concentrations of mannitol (n=4-6/group).

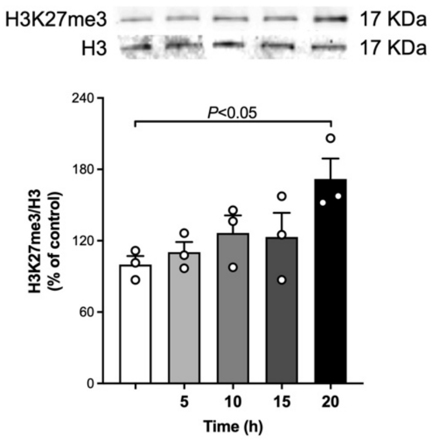

Fig. S3 Time-course of high glucose concentration and H3K27me3 expression. Representative western blot images and relative densitometric quantifications showing H3K27me3 protein in HAEC exposed to high glucose (25 mmol/l). The open bar represents H3K27me3 in cell exposed to normal glucose (5 mmol/l, n=3/group).

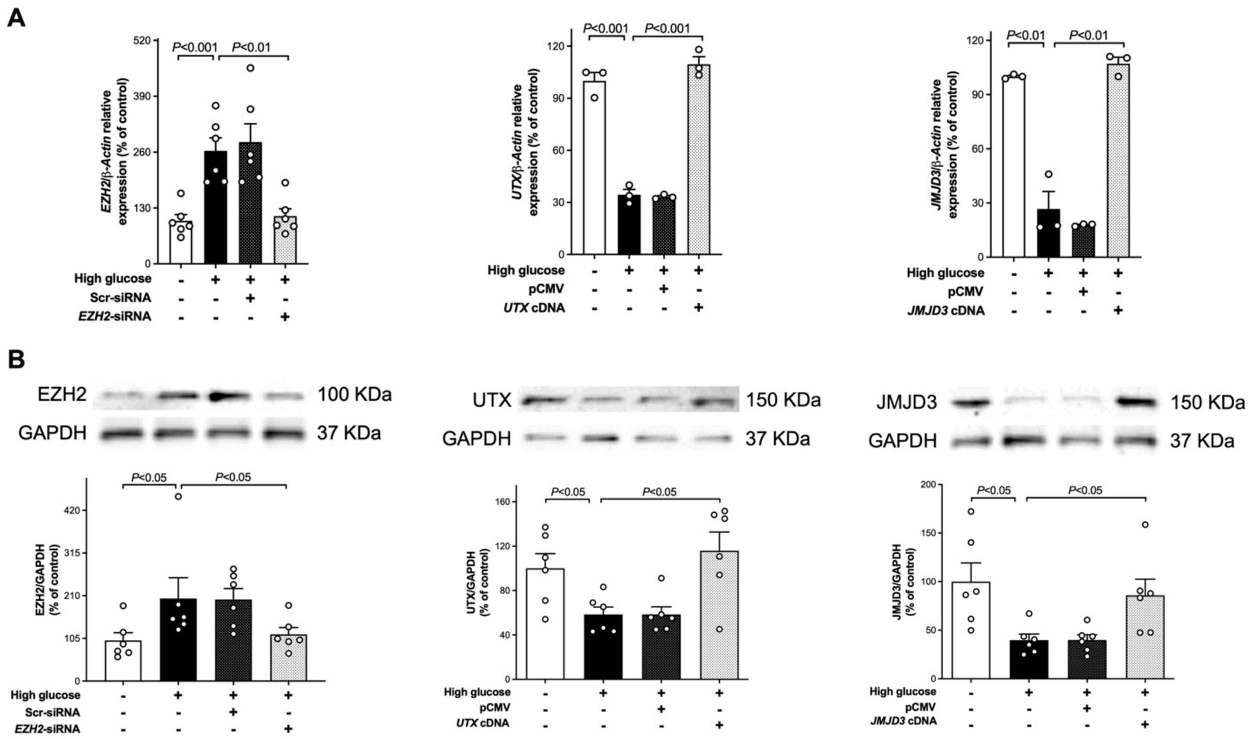

Fig. S4 Reprogramming of chromatin modifying enzymes by EZH2 siRNA, and UTX or JMJD3 overexpressing vectors. (A) mRNA and **(B)** protein expression of EZH2, UTX, and JMJD3 in HAEC exposed to normal (5 mmol/l) and high glucose (25 mmol/l). Scramble-siRNA and pCMV were used as controls for siRNA-mediated knockdown and vector-based overexpression, respectively (n=3-6/group).

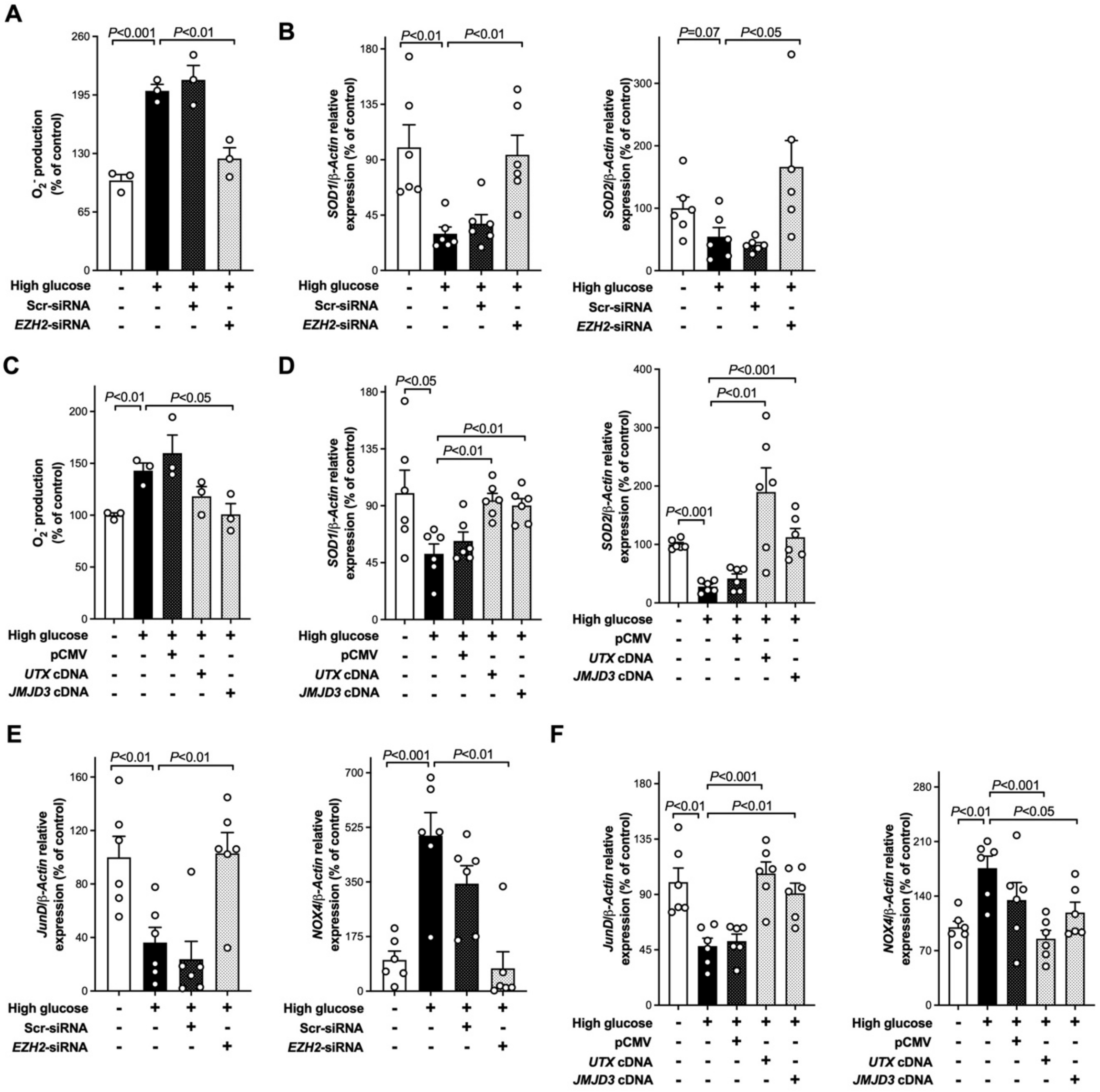

Fig. S5 Reprogramming of EZH2, UTX and JMJD3 expression levels blunts glucose-induced oxidative stress. **(A, C)** O_2^- production in the absence and in the presence of EZH2-siRNA, UTX cDNA, and JMJD3 cDNA, respectively (n=3/group). **(B and D-F)** Gene expression of *SOD1, SOD2*, *JunD* and *NOX4* in HAEC exposed to normal (5mmol/l) or high glucose in the presence and in the absence of EZH2-siRNA, UTX or JMJD3 overexpressing vectors. Scramble-siRNA and pCMV were used as controls for siRNA-mediated knockdown and vector-based overexpression, respectively (n=6/group).

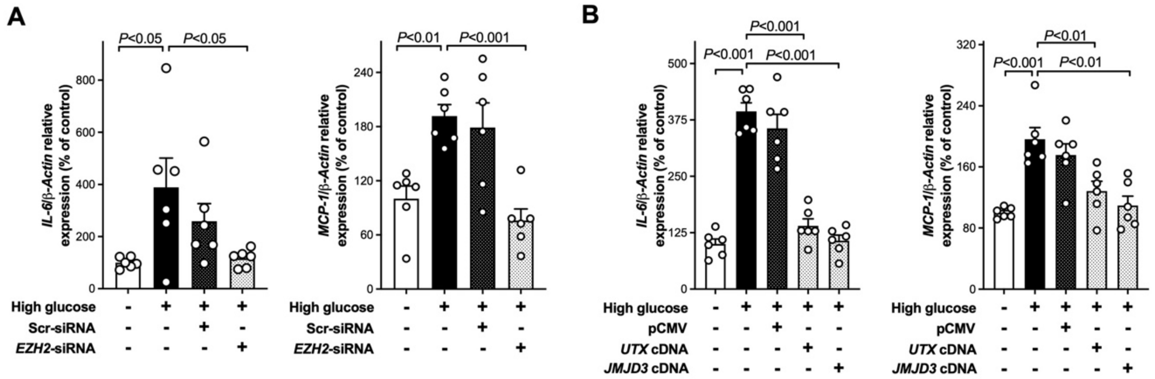

Fig. S6 Reprogramming of EZH2, UTX and JMJD3 expression abolishes glucose-induced inflammation. RT-qPCR showing *IL-6* and *MCP-1 gene expression* in HAEC exposed to normal (5 mmol/l) or high glucose (25 mmol/l) in the presence and in the absence of **(A)** EZH2 siRNA, and **(B)** UTX and JMJD3 overexpressing vectors. Scramble-siRNA and pCMV were used as controls for siRNA-mediated knockdown and vector-based overexpression, respectively (n=6/group).

Table S1. Primers used in RT-qPCR experiments.

	Primer sequences (5′ → 3′)	
**Gene**	**Forward**	**Reverse**
β-Actin	GTTGTCGACGACGAGCG	GCACAGAGCCTCGCCTT
ALDH1	CCACTCACTGAATCATGCCA	GCACGCCAGACTTACCTGTC
ALDH2	ACAATGGCAAGCCCTATGTC	ACAGGTTCATGGCGTGTGTA
CAT	AGTCAGGGTGGACCTCAGTG	CTGGAGAAGTGCGGAGATTC
EZH1	ACCATCGGATTGGGATCTTT	GCATCAGCTTGGCTGTACCT
EZH2	GGACTCAGAAGGCAGTGGAG	CCCTTCTCAGATTTCTTCCCA
GPX1	TTGACATCGAGCCTGACATC	ACTGGGATCAACAGGACCAG
ICAM-1	TCACACTGACTGAGGCCTTG	GGCTGGAGCTGTTTGAGAAC
IL-6	ACTCACCTCTTCAGAACGAATTG	CCATCTTTGGAAGGTTCAGGTTG
JunD	ATCGACATCGACACGCAGGAG	CTCCGTGTTCTGACTCTTCAGG
UTX	AATTCTGGGAGGAGGAGGAA	ATACGACACGCACAAAAGCA
JMJD3	AGACAGGGCACACCAAACTC	AGTCCTTTCACAGCCAATTCC
UTY	GAGCTGGAATGCAATGGTG	TGAGGTCAGGAGTTCAAGACAAG
MCP-1	AGCAAGTGTCCCAAAGAAGC	TGGAATCCTGAACCCACTTC
NOX4	GGATAAGGCTGCAGTTGAGG	AACCAAGGGCCAGAGTATCA
SOD1	CCACACCTTCACTGGTCCAT	CTAGCGAGTTATGGCGACG
SOD2	GCTCCGGTTTTGGGGTATCTG	GCGTTGATGTGAGGTTCCAG
TNFα	CAGCGCTGAGTCGGTCACCC	AGCCGCATCGCCGTCTCCTA
VCAM-1	TAAAATGCCTGGGAAGATGG	GGTGCTGCAAGTCAATGAGA
JunD	AATAACTCCTTGGCGCCTCC	CCAAGCGCTTTCAGCAACTT
NOX4	GATAAAGAAACTGGCGGCTG	GTAACGAAATTTGAGCCGGA
SOD1	AATCCTTGGCCCGAAAACCC	CAGCCAGCCCAGGAACGCAG
SOD2	TAAGCCGACCTTGGGACCTA	CAAAACATGACTGCCAGGGC

Table S2. Primers used in ChIP-qPCR assays.

	Primer sequences (5′ → 3′)	
**Gene**	**Forward**	**Reverse**
JunD promoter	AATAACTCCTTGGCGCCTCC	CCAAGCGCTTTCAGCAACTT
NOX4 promoter	GATAAAGAAACTGGCGGCTG	GTAACGAAATTTGAGCCGGA
SOD1 promoter	AATCCTTGGCCCGAAAACCC	CAGCCAGCCCAGGAACGCAG
SOD2 promoter	TAAGCCGACCTTGGGACCTA	CAAAACATGACTGCCAGGGC
